# Supplementary figures and images for: The rapidly evolving X-linked MIR-506 family fine-tunes spermatogenesis to enhance sperm competition
Source: eLife. 2024 Apr 19;13:RP90203. doi: 10.7554/eLife.90203 (PMC11031087; doi:10.7554/eLife.90203)

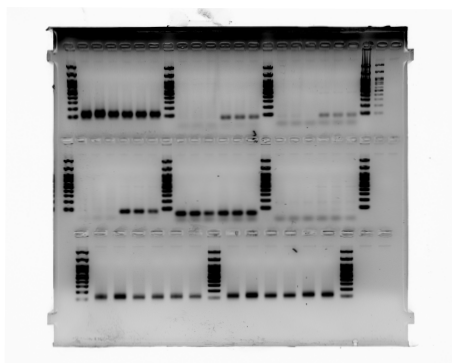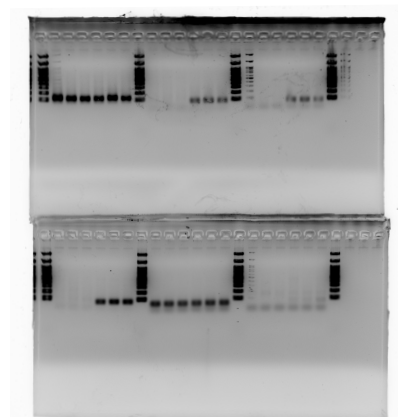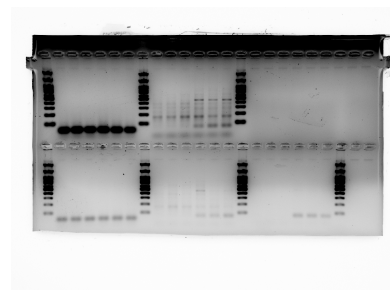

Supplement: Figure 2—source data 1. [file elife-90203-fig2-data1.zip › Figure 2-source data 1.pdf]

E

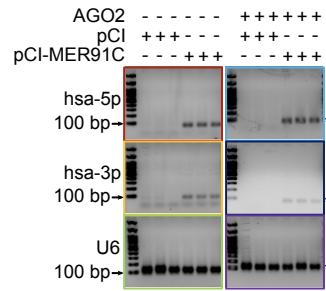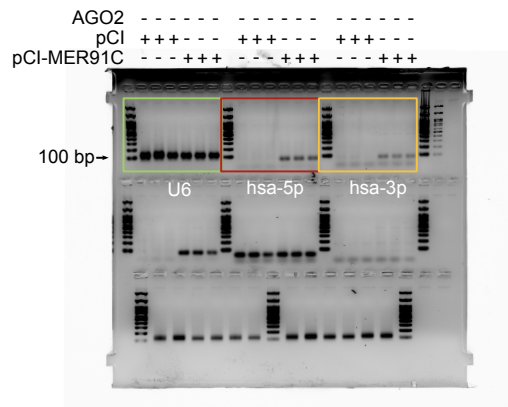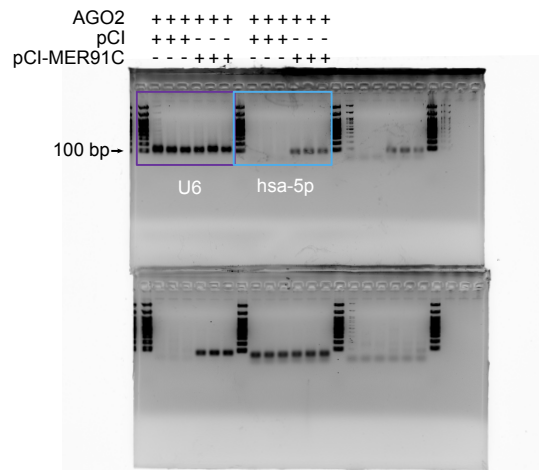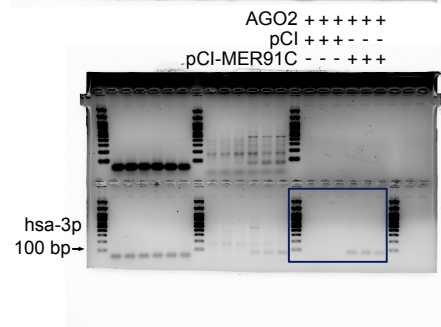

Supplement: Figure 2—source data 2. [file elife-90203-fig2-data2.zip › Figure 2-source data 2.pdf]

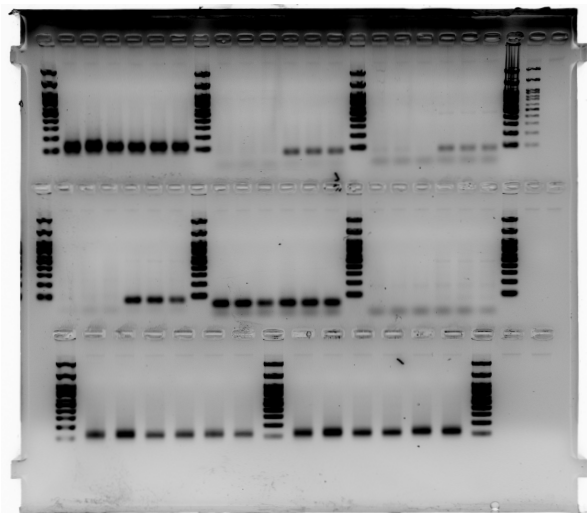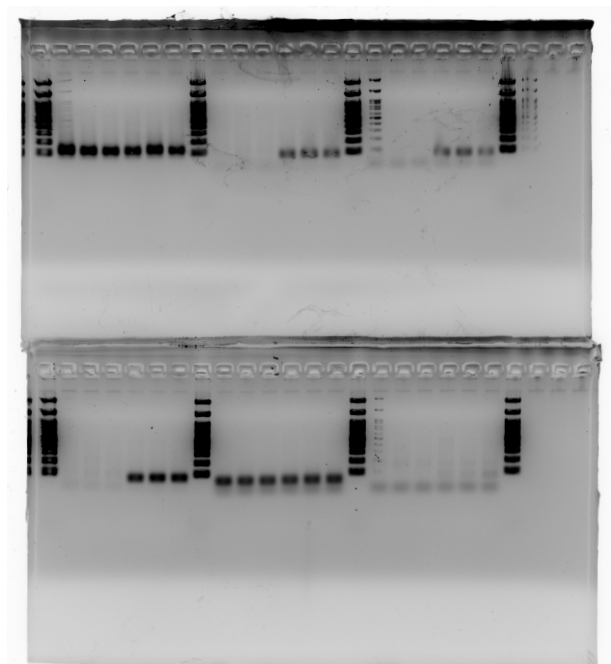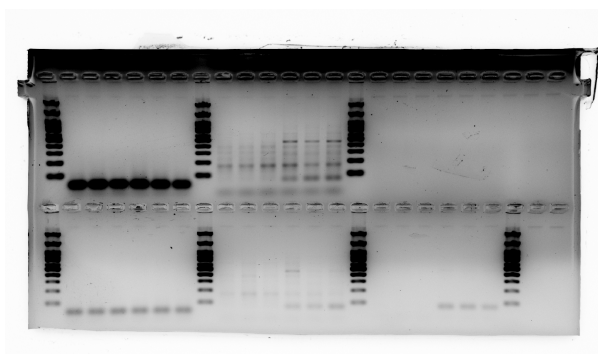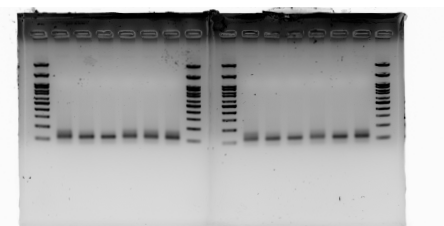

Supplement: Figure 2—figure supplement 3—source data 1. [file elife-90203-fig2-figsupp3-data1.zip › Figure 2-figure supplement 3-source data 1.pdf]

B

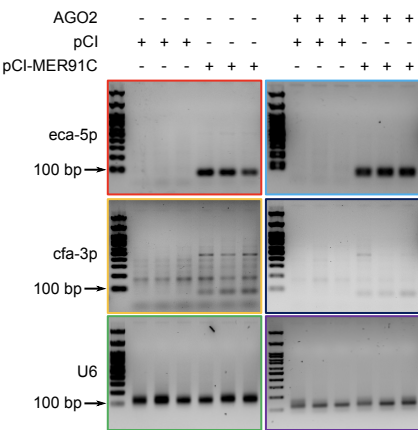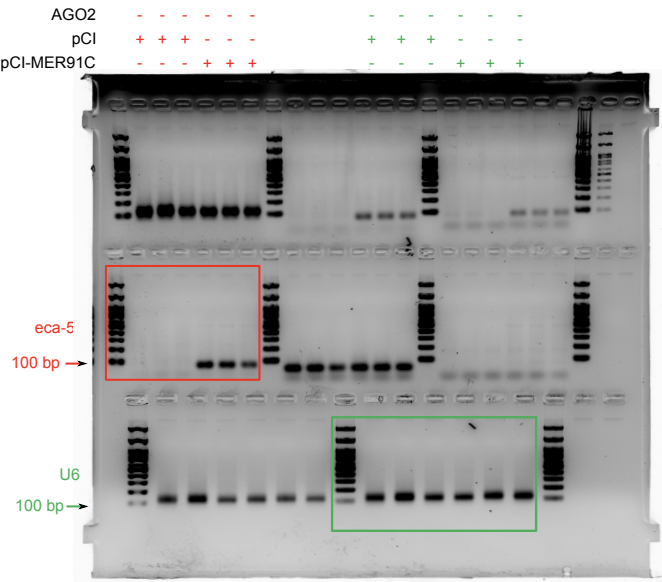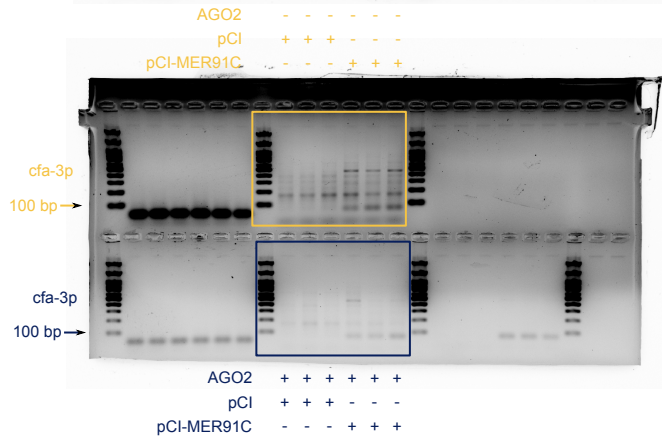

|    | AGO2 | pCI | pCI-MER91C |
|----|------|-----|------------|
| 1  | +    | +   | -          |
| 2  | +    | +   | -          |
| 3  | +    | +   | -          |
| 4  | +    | +   | -          |
| 5  | +    | +   | -          |
| 6  | -    | -   | +          |
| 7  | -    | -   | +          |
| 8  | -    | -   | +          |
| 9  | -    | -   | +          |
| 10 | -    | -   | +          |

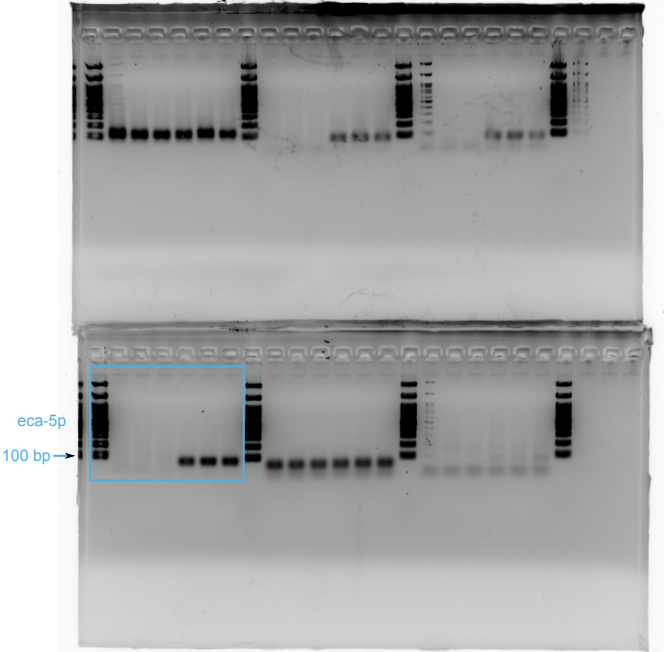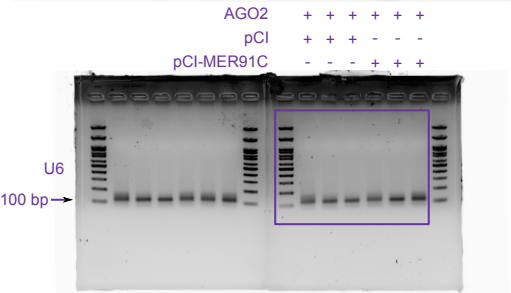

Supplement: Figure 2—figure supplement 3—source data 2. [file elife-90203-fig2-figsupp3-data2.zip › Figure 2-figure supplement 3-source data 2.pdf]

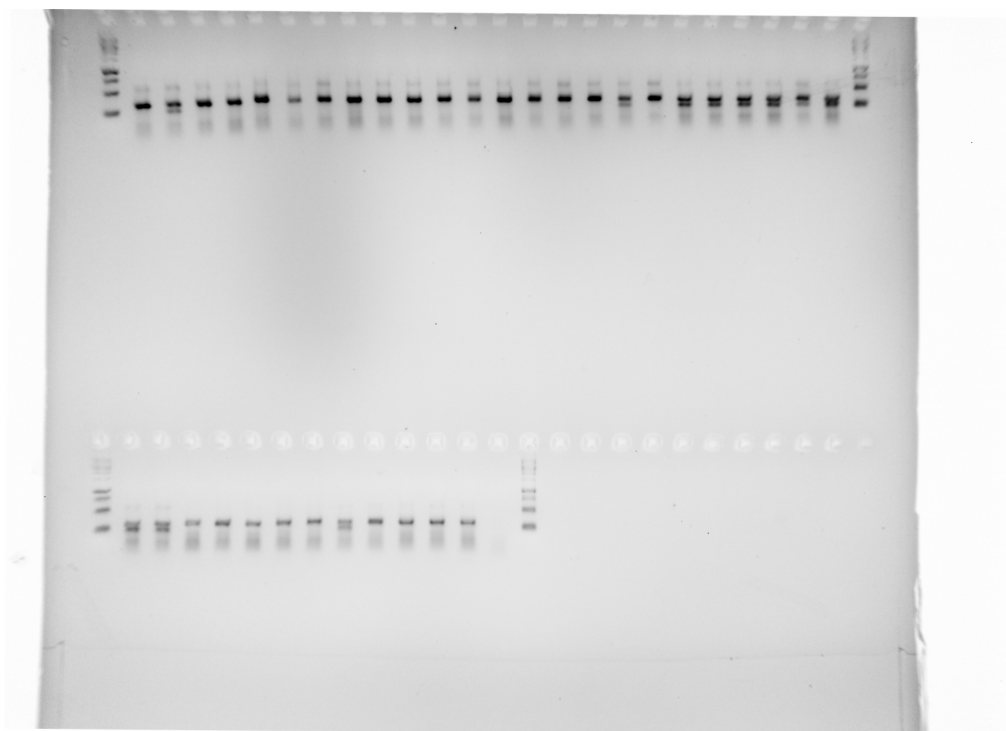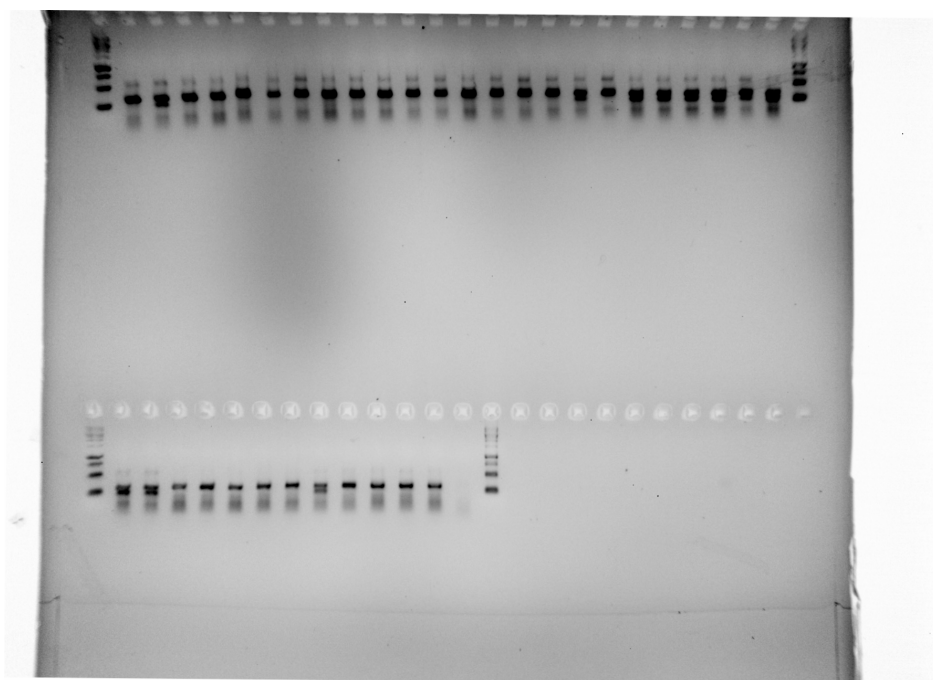

Supplement: Figure 4—source data 1. [file elife-90203-fig4-data1.zip › Figure 4-source data 1.pdf]

H

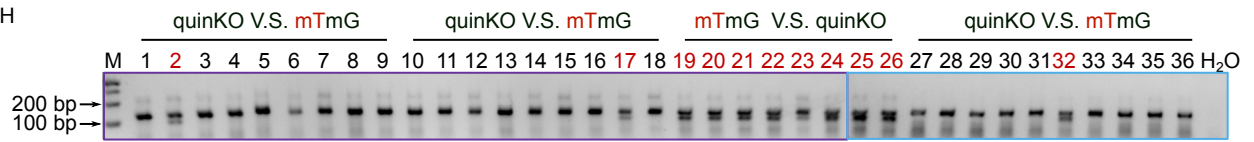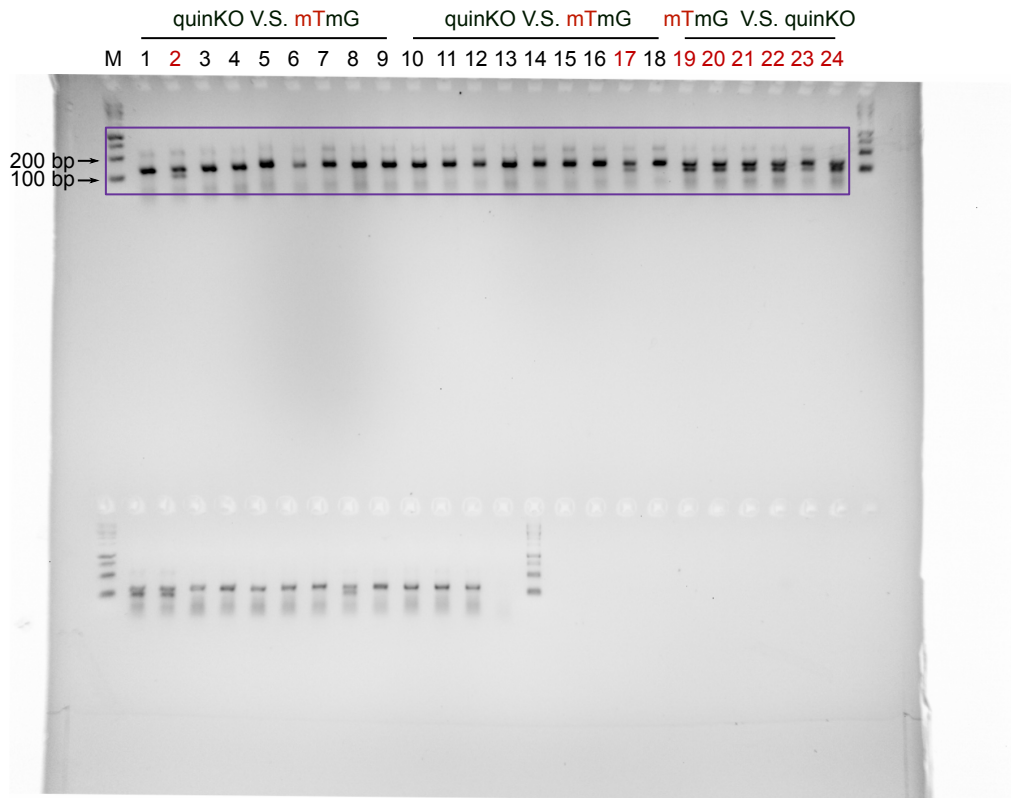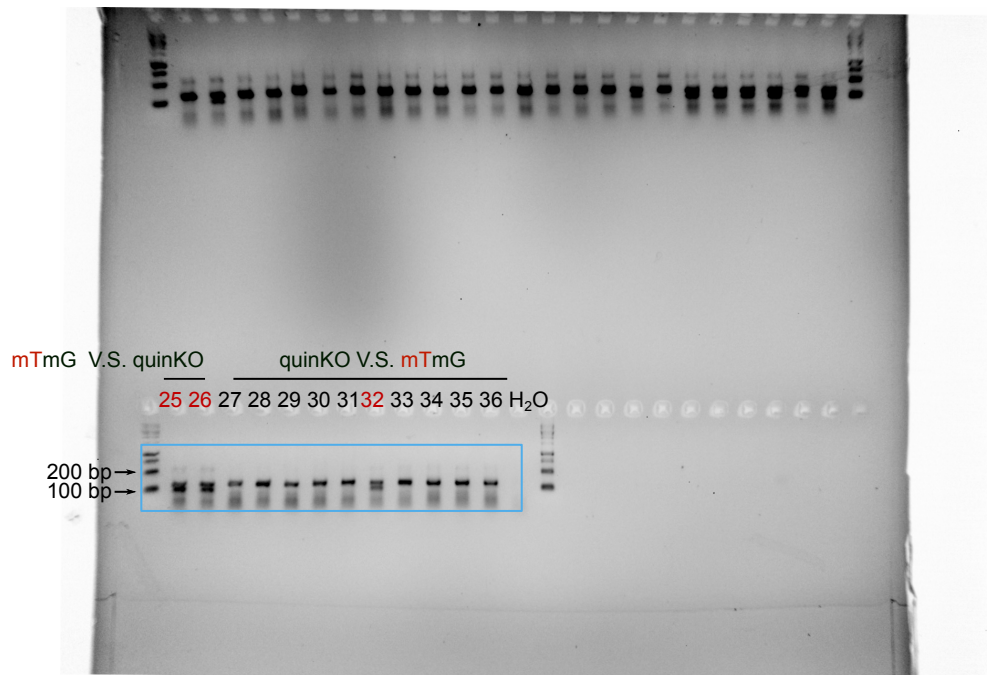

Supplement: Figure 4—source data 2. [file elife-90203-fig4-data2.zip › Figure 4-source data 2.pdf]

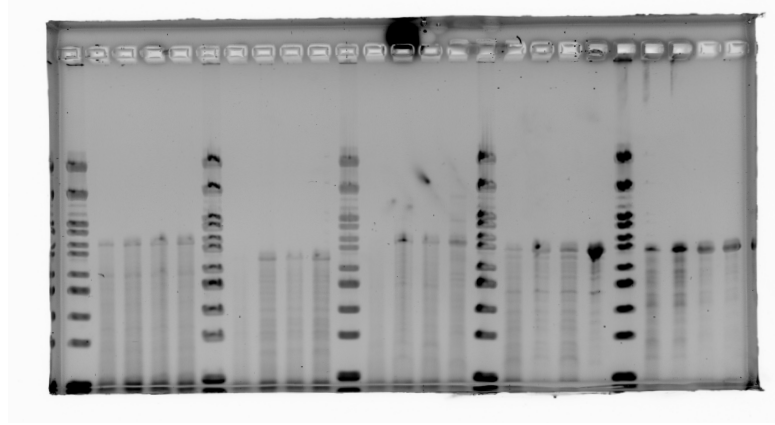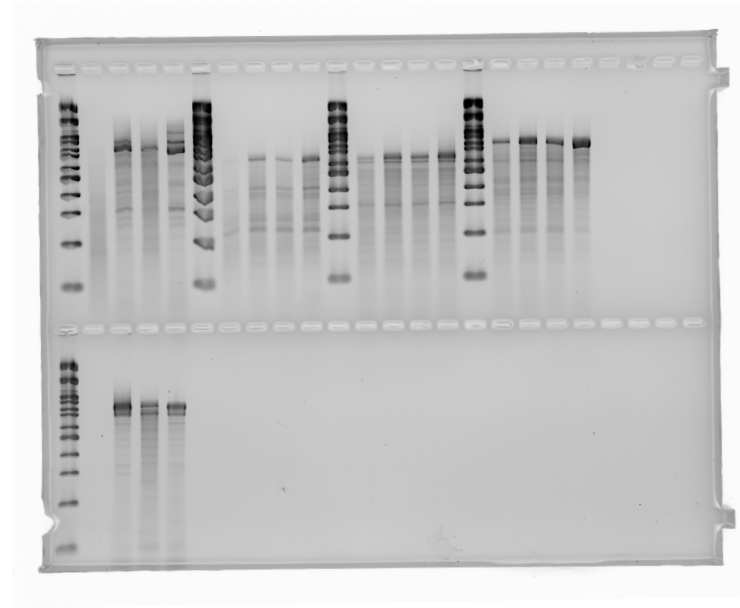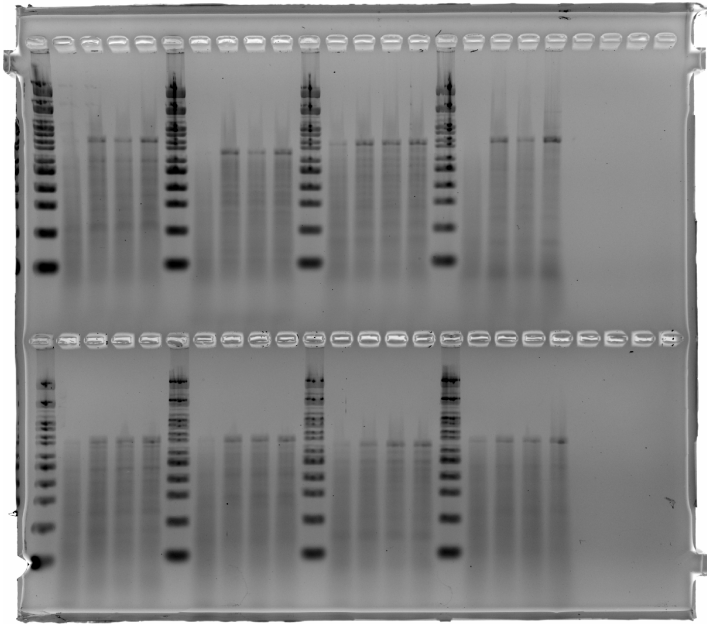

Supplement: Figure 4—figure supplement 1—source data 1. [file elife-90203-fig4-figsupp1-data1.zip › Figure 4-figure supplement 1 source data 1.pdf]

F

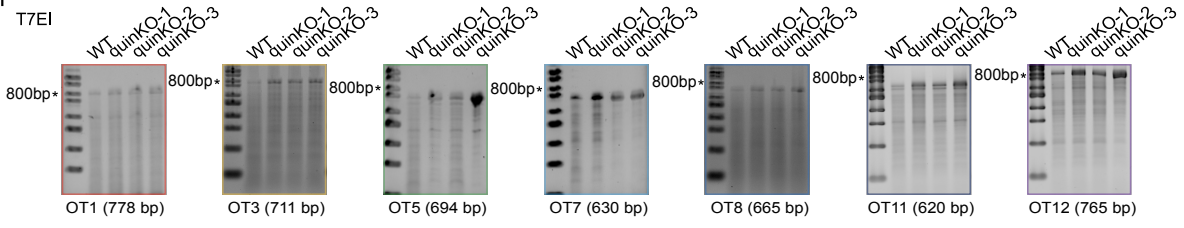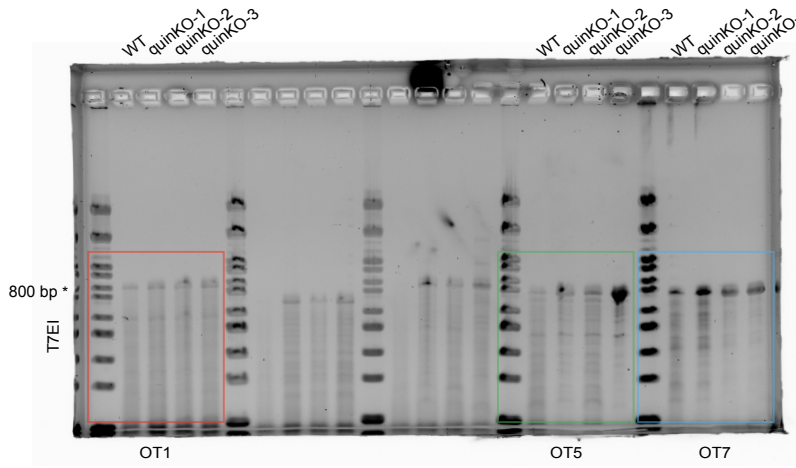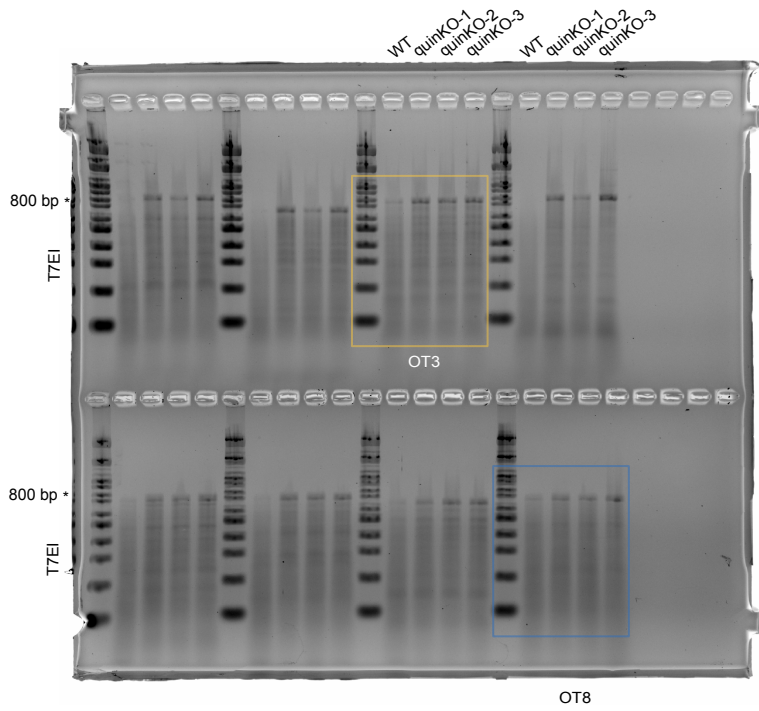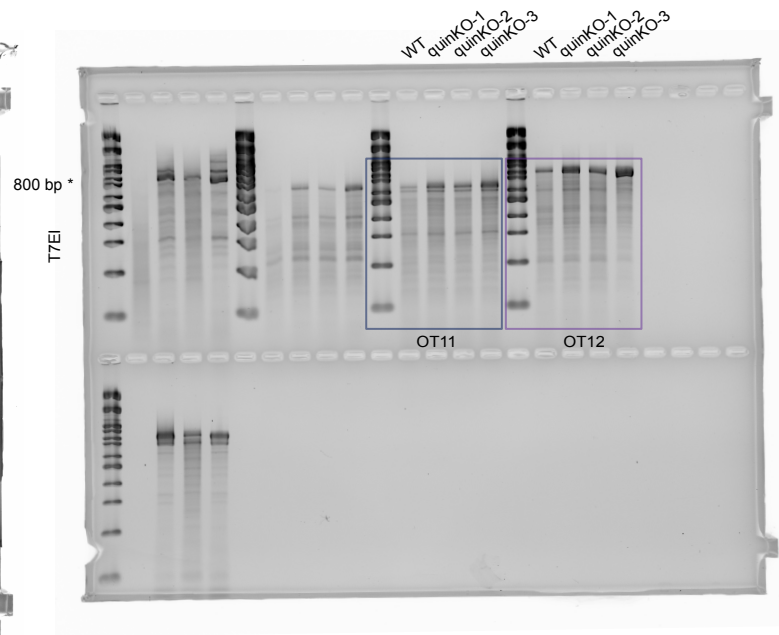

Supplement: Figure 4—figure supplement 1—source data 2. [file elife-90203-fig4-figsupp1-data2.zip › Figure 4-figure supplement 1 source data 2.pdf]

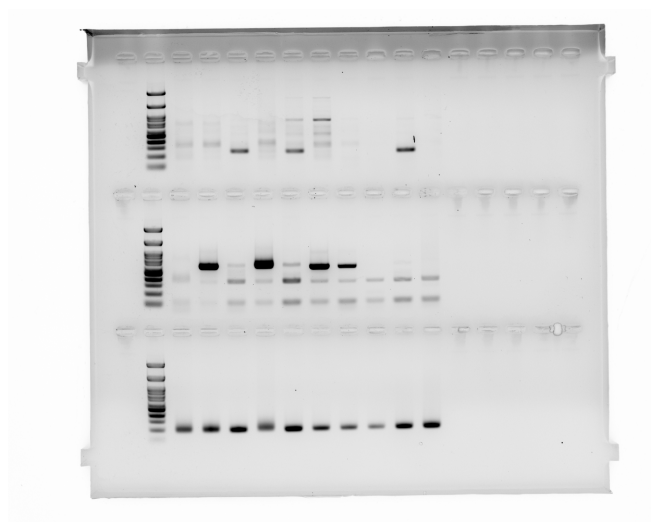

Supplement: Figure 4—figure supplement 1—source data 3. [file elife-90203-fig4-figsupp1-data3.zip › Figure 4-figure supplement 1 source data 3.pdf]

J

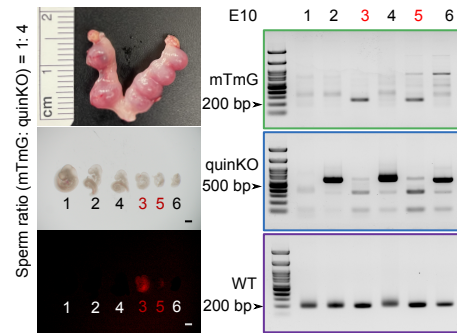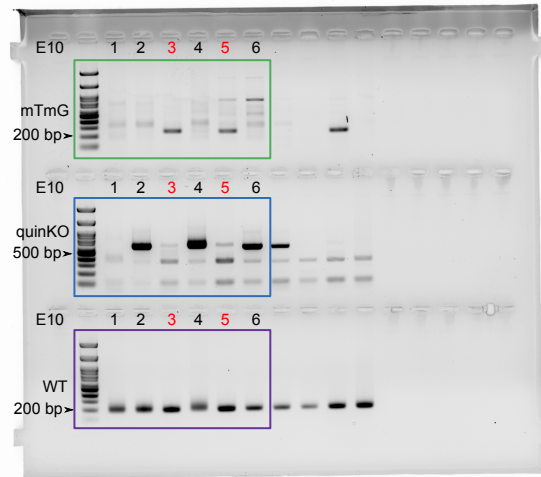

Supplement: Figure 4—figure supplement 1—source data 4. [file elife-90203-fig4-figsupp1-data4.zip › Figure 4-figure supplement 1 source data 4.pdf]

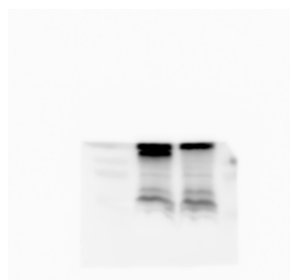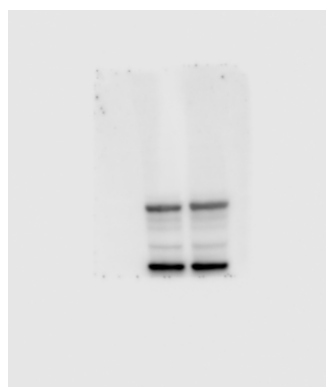

Supplement: Figure 5—figure supplement 1—source data 1. [file elife-90203-fig5-figsupp1-data1.zip › Figure 5-figure supplement 1-source data 1.pdf]

E

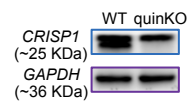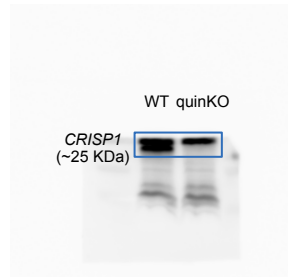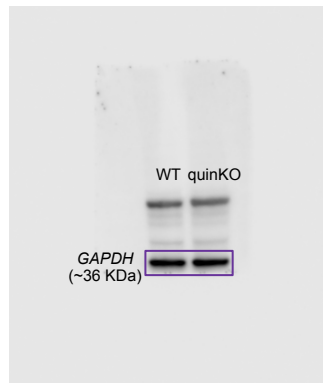

Supplement: Figure 5—figure supplement 1—source data 2. [file elife-90203-fig5-figsupp1-data2.zip › Figure 5-figure supplement 1-source data 2.pdf]
